# Higher coactivations of lower limb muscles increase stability during walking on slippery ground in forward dynamics musculoskeletal simulation

**DOI:** 10.1038/s41598-023-49865-w

**Published:** 2023-12-20

**Authors:** Young-Jun Koo, Jemin Hwangbo, Seungbum Koo

**Affiliations:** Department of Mechanical Engineering, Korea Advanced Institute of Science and Technology, 291 Daehak-ro, Yuseong-gu, Daejeon, 34141 Republic of Korea

**Keywords:** Musculoskeletal system, Biomedical engineering, Mechanical engineering

## Abstract

The energy efficiency theory of human bipedal locomotion has been widely accepted as a neuro-musculoskeletal control method. However, coactivation of agonist and antagonist muscles in the lower limb has been observed during various limb movements, including walking. The emergence of this coactivation cannot be explained solely by the energy efficiency theory and remains a subject of debate. To shed light on this, we investigated the role of muscle coactivations in walking stability using a forward dynamics musculoskeletal simulation combined with neural-network-based gait controllers. Our study revealed that a gait controller with minimal muscle activations had a high probability of falls under challenging gait conditions such as slippery ground and uneven terrain. Lower limb muscle coactivations emerged in the process of gait controller training on slippery ground. Controllers with physiological coactivation levels demonstrated a significantly reduced probability of falls. Our results suggest that achieving stable walking requires muscle coactivations beyond the minimal level of muscle energy. This study implies that coactivations likely emerge to maintain gait stability under challenging conditions, and both coactivation and energy optimization of lower limb muscles should be considered when exploring the foundational control mechanisms of human walking.

## Introduction

Skeletal muscles surrounding joints consist of agonist and antagonist muscles^1^. The agonists and antagonists generate joint torques in opposite directions. Simultaneous activation of these opposing muscles, termed muscle coactivation, is often observed during various movements, such as walking^2–4^. Yet, the underlying mechanism for the emergence of coactivation remains poorly understood^5^. Theories promoting energy-optimal human movement theories are widely accepted as explanations for human movement behaviors^6^ and the mechanisms behind how humans control their movements^7,8^. According to the theory of energy-efficient walking, human skeletal muscles should be coordinated to reduce metabolic energy during walking^7,9^. However, this theory struggles to account for the observed emergence of coactivation during movements^5^.

The phenomenon of muscle coactivation during walking has been investigated through muscle synergies^2,4^ by quantifying muscle synergy vectors^9,10^. Hogan^1^, Burdet et al.^11^, and Latash^12^ suggested that muscle coactivation increases joint stiffness. The increased joint stiffness may help maintain joint stability complemented by ligament tension and joint surface contact during movements^13^. High levels of coactivations have been observed when the probability of destabilization, such as slips and force perturbations, increased during walking^14^. Moreover, Voloshina et al.^15^ reported an increase in coactivation during walking on uneven terrain and Muller et al.^16^ reported an increase in muscle activations of both the gastrocnemius and tibialis anterior under step-down perturbation conditions. However, Hortobagyi et al.^3^ and Nagai et al.^5^ reported that large coactivation of the lower limb muscles diminishes the performance of agonist muscles and ability to adapt to perturbation. The onset of muscle coactivation can be influenced by psychological factors, such as the fear of falling, which may disrupt the phasic neural control system^17,18^. Understanding the origins and functions of physiological coactivations between agonist and antagonist muscle pairs during walking can shed light on the core neuro-musculoskeletal control strategies of the lower limbs.

Muscle functions and dynamics during walking can be studied using dynamics simulation of musculoskeletal models. The inverse dynamics simulation^19^ determines joint torques, muscle contraction forces, and intra-articular contact forces from measured body motions and external forces, such as gravity and ground reaction forces. These simulations assist in understanding injury mechanisms, including ligament tears and degenerative joint injuries^20^. However, human joints are mechanically redundant, meaning there are infinite combinations of muscle forces that can produce a particular movement. As a result, the muscle forces or activation levels in the inverse dynamics simulation are determined by minimizing a cost function, often represented as the sum of squared muscle activation levels, although other exponents have also been investigated^21–24^, in accordance with the energy-optimal human movement theory^7,9^. However, the energy consumption minimization approach struggles to reproduce physiological muscle coactivations^25,26^. Antagonistic contraction can hinder the generation of joint moments in the desired direction of joint motions and can increase the cost of the objective function. Therefore, the energy minimization method often underestimates antagonist coactivation^12,26^. Attempts to incorporate physiological coactivation phenomena in simulations have persisted in electromyography-driven musculoskeletal simulations^27^ and muscle synergy vector-based simulations^28^. Nonetheless, there’s still a gap in our understanding of the emergence of muscle coactivation.

Over the past decade, research in artificial intelligence has seen rapid advancements. One of the standout techniques, Reinforcement learning (RL), excels at determining control strategies for high-dimensional dynamic systems^29,30^. It is possible to create a participant-specific gait controller for forward dynamics gait simulation by imitating a participant’s walking motion^31,32^. Computational competitions, such as “Learning to Run,” “Artificial Intelligence for Prosthetics,” and “Learn to Move: Walk Around” were hosted at the Conference on Neural Information Processing Systems in 2017, 2018, and 2019, respectively. These competitions promoted RL research for musculoskeletal simulations, covering both physiological (e.g. walking and running) and pathophysiological (e.g. walking with prosthetics) conditions^33–35^.

While traditional optimization techniques have provided invaluable insights into understanding and predicting human movement, they often center on finding the optimal solution for a specific scenario^36,37^. Once optimized, these solutions may not easily adapt to changing conditions. In contrast, RL offers a novel approach in this realm. Rather than optimizing for a singular event, RL aims to develop a control policy by maximizing the expected cumulative reward over time. This results in a learned policy that, once trained, can be applied across diverse scenarios, offering adaptability and generalization.

In this study, we leverage the power of RL to produce control policies that can be efficiently employed in forward simulations across different walking terrains and conditions, highlighting its potential in biomechanics and robotics research. Nowakowski et al.^38^ endeavored to harness the RL technique for two-dimensional human locomotion. They utilized bio-inspired reward shaping strategies and studied muscle activation patterns in a two-dimensional musculoskeletal model with eight degrees-of-freedom and 22 muscles. Forward dynamics simulations employing RL-based muscle controllers enable a deep dive into the neuro-muscular control mechanisms underpinning human movements.

In this study, we hypothesized that the elevated coactivation of agonist–antagonist muscles, or increased joint stiffness, in the lower limb enhances stability during walking under challenging conditions, such as on slippery or uneven terrains. To test these hypotheses, we used RL-based gait controllers for a human model and forward dynamics simulations that reproduce a participant’s walking motion but with varying degrees of muscle coactivations and energy consumptions. Walking stabilities were quantified by counting the number of survivals when the human model walked on slippery or uneven terrains.

The frequency and incidence of falls serve as indicators of stability during daily activities such as walking^39,40^. Nonetheless, carrying out falling experiments during human walking is problematic due to ethical and safety concerns^41,42^. The forward dynamics-based gait simulations in this study were designed to reproduce and simulate falls during walking under challenging conditions.

## Results

### Gait simulation using gait controllers

**Figure 1:**
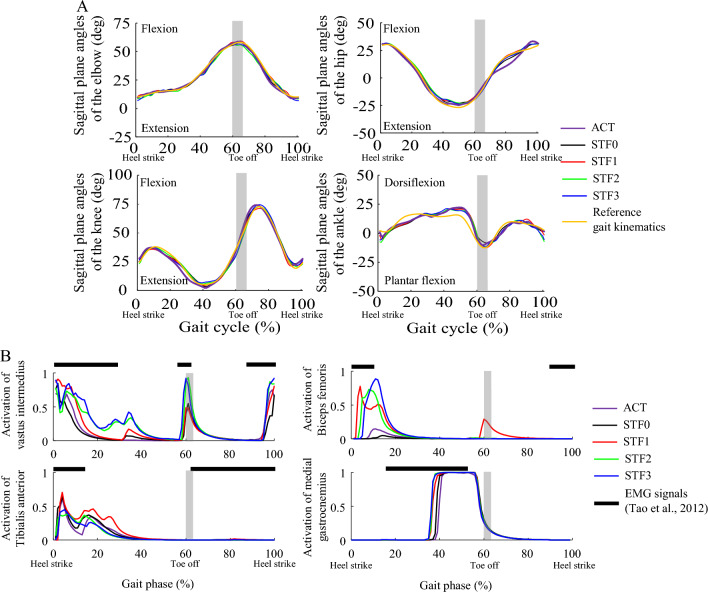
(**A**) Sagittal plane angles of the lower limb and elbow joints during walking simulation using five different gait controllers; and (**B**) muscle activation patterns of the five controllers, along with on/off diagrams of electromyography signals^34^ for lower limb muscles. The grey vertical bars indicate toe off.

The standard gait controller, trained using deep RL-based imitation learning, exhibited a high gait kinematics score, ${r}_{track}$, of 0.975. This suggests that the temporal patterns of joint angles closely matched those of the reference motion data. The five fine-tuned controllers — denoted as ACT, STIF0, STIF1, STIF2, and STIF3 — also achieved gait kinematics scores ranging from 0.973 to 0.975 (Fig. 1A). There was no statistically significant difference according to the analysis of variance test at a significance level of 0.05. All the fine-tuned controllers were able to generate stable walking motions without any falls on normal ground condition with a friction coefficient of 0.8. The root-mean-squared differences of the joint angles between the reference motions and simulated motions ranged from 1.3° to 9.6° for the five fine-tuned controllers (Table 1).

**Table 1:**
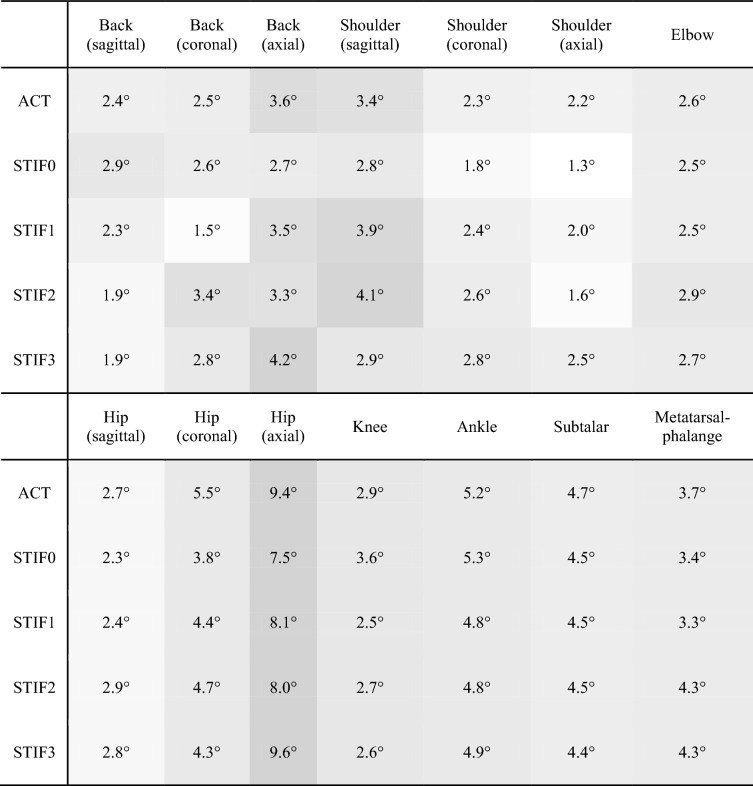
Root-mean-squared difference of all body joints between walking kinematics simulated using five different gait controllers and inverse kinematics results of measured human kinematics using OpenSim.

The five fine-tuned controllers showed different muscle activation patterns, even though the gait kinematics were very close to each other. Temporal muscle activation patterns in our results showed similar trends, but the activation levels of the lower limb muscles differed among the five controllers (Fig. 1B). The activation timing of the vastus intermedius aligned with the electromyography results from a previous study^43^. The biceps femoris and tibialis anterior were activated only at the beginning of the stance phase, despite the electromyography results showing activation during the swing phase^43^. For the gastrocnemius, the simulated activation results lagged behind the electromyography findings^43^. ACT and STIF0 used the thigh muscles less than STIF1, STIF2, and STIF3. Meanwhile, STIF2 and STIF3 used the tibialis anterior less than the other controllers.

### Muscle coactivation index

**Figure 2:**
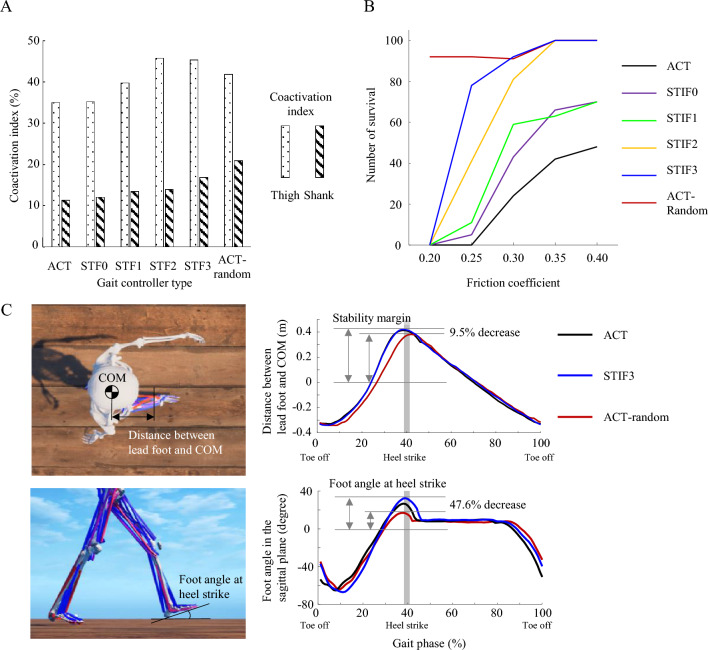
(**A**) Coactivation indices of the thigh and shank during gait simulation on flat ground with a normal friction coefficient; (**B**) the number of walking simulation survivals over a 20-s period on a slippery ground without fall; and (**C**) stability margin and foot angle at heel strike during gait simulation on flat ground with a normal friction coefficient.

Using analysis of variance at a significance level of 0.05, we found statistically significant differences in the muscle coactivation indices of the thigh and shank among the five controllers during gait simulations on flat ground with a normal friction coefficient 0.8, except between ACT-STIF0 and STIF2-STIF3. The averages (± standard deviations) of the thigh coactivation indices were 35. 0 (± 1.4), 35.2 (± 1.0), 39.8 (± 0.9), 45.8 (± 1.0), and 45.3 (± 1.6) and those of the shank coactivation indices were 11.3 (± 1.3), 12.0 (± 0.3), 13.5 (± 0.4), 14.0 (± 0.9), and 16.9 (± 1.2) for the ACT, STIF0, STIF1, STIF2, and STIF3, respectively (Fig. 2A). Among the controllers, the lowest values of thigh and shank coactivation indices were observed for the ACT. As the target joint stiffness values of the lower limb joints increased during fine-tuning of the controller, both the thigh and shank coactivation indices rose. The joint stiffness of the gait controllers similarly increased with higher stiffness target values. The average stiffness of the knee joint was 8.0 Nm/degree for ACT, 6.6 Nm/degree for STIF0, 8.6 Nm/degree for STIF1, 11.6 Nm/degree for STIF2, and 12.9 Nm/degree for STIF3, respectively. Although STIF0 was trained to minimize joint stiffness, its stiffness was greater than zero during walking.

### Walking simulation on slippery ground

Each controller underwent 100 tests, each lasting 20 s, on five slippery ground conditions with friction coefficients ranging from 0.20 to 0.40. The number of successful trials (no falls) of all the controllers are summarized in Table 2A. There was a statistically significant difference in the performance of the six gait controllers across the various walking conditions according to the Chi-squared test. Based on the post-hoc pairwise Chi-squared tests, the performances were ranked as follows: ACT < STIF0 = STIF1 < STIF2 < STIF3 < ACT-random. The survival count decreased as the friction coefficient of the ground decreased for all controllers. Among the five controllers, the ACT recorded the fewest survivals under all slippery ground conditions, having no survivals at friction coefficient of 0.20 and 0.25. The number of survivals rose when the controller was fine-tuned to larger target stiffness values for the lower limb joints.

**Table 2:**
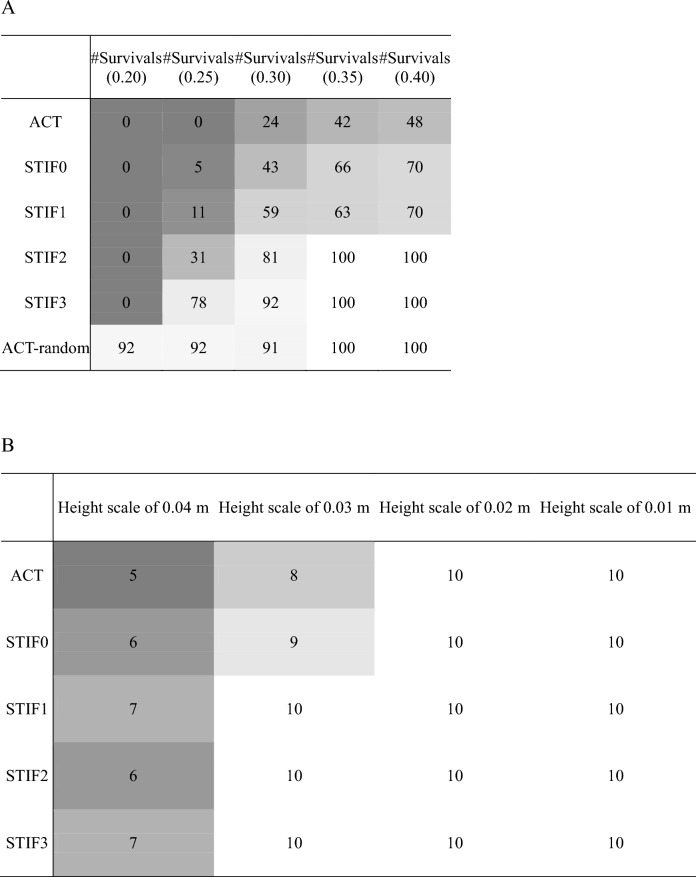
(A) Number of survivals out of 100 repetitions of 20-s walking simulations and (B) and the number of survivals out of 10 repetitions of 20-s walking simulations on uneven terrains.

The ACT-random had the highest survival numbers for all ground friction conditions (Fig. 2B). The ACT-random had larger muscle coactivation indices in the thigh and shank than those of the ACT (Fig. 2A), even though ACT-random had the same objective function as that of the ACT. In both cases, the distance between the body’s center of mass and the lead foot in the transverse plane, as well as the foot angle in the sagittal plane at heel strike, showed statistically significant decreases compared to those of ACT and STIF3, as determined by analysis of variance (Fig. 2C). The percentage decreases in the distance and foot angle were 9.5% and 47.6%, respectively, compared to STIF3.

### Walking simulation on uneven terrain

The number of survivals out of 10 repetitions for walking tests on uneven terrain is summarized in Table 2B. On uneven terrains with height scales 0.03 m and 0.04 m, ACT recorded the fewest survivals, while STIF3 had the highest survival count. For terrains with height scales of 0.02 m or less, all controllers completed all 10 repetitions without falls. However, there was no statistically significant difference in survival rates among the five controllers when considering all conditions collectively or when focusing solely on height scale of 0.03 m.

## Discussion

The forward dynamics gait simulations were conducted using gait controllers with varying levels of muscle coactivations to test the hypotheses. Our results support the hypothesis that the elevated coactivation of agonist–antagonist muscles, or increased joint stiffness, in the lower limb enhances stability during walking under challenging conditions, such as on slippery or uneven terrains. When the surface friction ranged from 0.2 to 0.4, the ACT — which aimed to minimize the sum of squared muscle activation (a marker of energy-efficient walking) — exhibited the lowest survival rate at 22.8%, as shown in Table 2A. In contrast, the survival rates for STIF1, STIF2, and STIF3 were 40.6%, 62.4%, and 74.0%, respectively. The ACT had a lower stability performance, specifically the number of survivals during walking, than that of STIF1, STIF2, and STIF3 on slippery ground. In other words, the gait controller that pursued only energy-efficient walking^7,9^ was less stable in challenging ground conditions (Table 2A), and the increase in joint stiffness and coactivations may occur in such walking conditions. Muscle coactivation and joint stiffness of the lower limbs play important roles in increasing gait stability when walking in challenging ground conditions although they increase energy consumption^27^.

Our results showed that large coactivations in the lower limbs would be required to prevent fall when walking on slippery ground. In previous studies, population who felt dynamic instability during walking, such as elderly people^4^ and joint disease patients^44,45^, had more coactivation during walking than healthy young population although atrophied muscles of elderly could decrease the effect of co-activations. Meanwhile, certain studies reported that the high coactivations in populations with dynamic instability might result from fear of falls and change in the neuro-control system^5,46^. According to the results of the STIF0, STIF1, STIF2, and STIF3, large joint stiffness and coactivation improved stability performances when walking on slippery and uneven ground conditions (Fig. 2A,B and Table 2). The number of survivals among the controllers were highest in STIF3 and lowest in STIF0 in slippery and uneven ground conditions. That is, the gait controller that generated large coactivations and joint stiffness in the lower limbs increased gait stability in challenging walking conditions.

**Figure 3:**
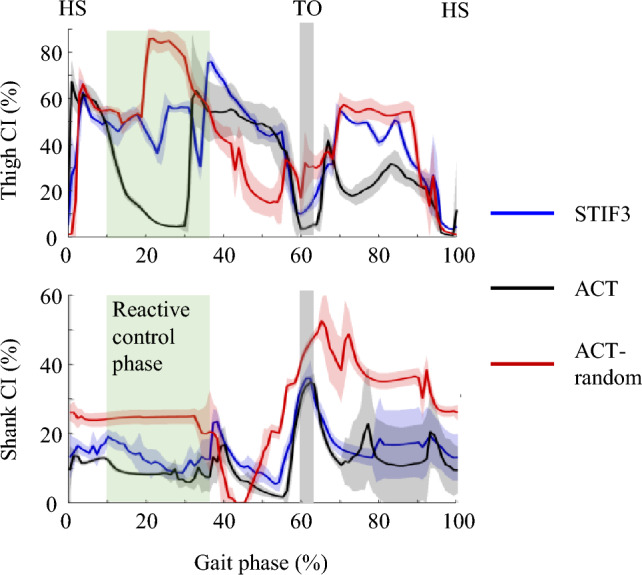
Temporal coactivation indices of the thigh and shank during musculoskeletal walking simulation.

During walking, slips of the foot on slippery ground typically occurred 100–400 ms after heel strike^47,48^. Reactive control, which is a control strategy employed after a slip to generate corrective movements^49^ was observed at this phase^50^. Temporal patterns of coactivations in ACT, STIF3, and ACT-random were different in the reactive control phase—marked as green blocks in Fig. 3. ACT-random had the largest coactivations and number of survivals in the reactive control phase, followed by STIF3. Madehkhaksar et al.^14^ suggested that large coactivations were associated with the improvement in the response time to generate corrective movements. Additionally, Wolf and Hirzinger^51^ discussed the benefits of motor control systems with heightened joint stiffness in responding to impacts and moving along fast trajectories. Our results showed that, when walking on the slippery ground, heightened coactivations during the reactive control phase conferred an advantage in terms of gait stability.

Neuro-control strategies for stable walking on slippery ground include proactive control, which occurs before a person encounters a potential slip^49,52^. As part of proactive strategies^48^, ACT-random not only increased coactivations in lower limb muscles but also decreased the stability margin and foot angle in the sagittal plane at heel strike (Fig. 2). The velocity of the body’s center of mass is often used to assess dynamic stability, such as the margin of stability^53,54^. However, the velocities of the body’s center of mass were very similar across different gait controllers in our study since they were all trained to imitate the same reference motion. Both the foot angle and stability margin were calculated to quantify the risk of falls^48,55^, especially during walks on slippery ground. The foot angle and margin of stability were the kinematic characteristics of ACT-random. While ACT-random was trained without a specific joint stiffness target, it exhibited increased coactivation and certain kinematic changes, particularly in foot angle and margin of stability. Such phenomena are observable in human walking on slippery ground, and our results align with experimental findings. The results demonstrated that proactive control strategies as well as large joint stiffness and coactivations should be accompanied to prevent falls when walking on a slippery ground. We tested the ACT-random only on the slippery ground. We would explore the ACT-random for uneven terrain in future studies.

The ACT had lower coactivation indices than the experimentally measured values of approximately 40 and 20 for the thigh and shank, respectively, using electromyography devices during normal walking of healthy young adults^4^. Energy-efficient walking produced relatively low levels of coactivation in musculoskeletal simulations. Therefore, ACT which seek to minimize the sum of squared muscle activations could not reproduce physiological coactivation levels. The coactivation levels observed in young adults during normal walking could be reproduced when a gait controller was trained to adjust joint stiffness of lower limbs. Although muscle synergy models^28^ and electromyography-driven musculoskeletal models^27^ have been investigated to reproduce muscle coactivations during musculoskeletal gait simulations, our research demonstrated that it could be possible to achieve coactivation modeling in musculoskeletal simulations without relying on these methods, simply by coordinating muscles to enhance joint stiffness, reflecting a neuro-control physiology.

During the musculoskeletal gait simulation, all gait controllers exhibited consistent gait kinematics scores. This suggests that these controllers could track the experimentally measured gait kinematics with a root mean squared error of less than 5.0°, with the exception of hip internal–external rotation and ankle dorsiflexion–plantarflexion. The gait kinematics score included position errors of the head, two hands, and two feet. These errors were not considerably affected by the hip internal–external rotation and might lead to the large root mean squared error of approximately 9.0°. In the case of the ankle joint, the ground contact calculation in RaiSim dynamics solver^56^ uses a hard contact formulation, which might not reflect the compliant nature of the contacts. This discrepancy could result in kinematics-tracking errors of ankle joints during the stance phase of walking.

In gait analysis, the minimum toe clearance was a crucial factor concerning gait stability^57^, especially when walking on uneven terrains. A variation in minimum toe clearance across different controllers could heighten the risk of falls. Nonetheless, our Chi-squared test did not reveal the significant difference in the survival rates between the gait controllers during walking on uneven terrains. Primarily, the ground height variations, particularly those limited to 0.04 m or less, could be insubstantial enough not to pose a considerable challenge to the controllers in terms of stability. Furthermore, the limited sample size chosen for the uneven terrain trials may have influenced the robustness and statistical significance of our findings.

The simulation on uneven terrain posed technical challenges because of unstable contact phenomena between the foot’s contact geometry and ground edges in the dynamics solver. It took a relatively long time to obtain valid trials in the uneven terrain, prompting us to reduce the number of tests compared to the sample size for slippery ground experiment. Another limitation is that the study was based on the gait data of a single subject. While this was caused by the relatively challenging task of training controllers in the RL-based forward dynamics gait simulation research, the results might not capture the variability in a larger population. For future work, it would be interesting to see how increased activation changes between subjects. Investigating the effects of different parameters such as body mass could provide a more comprehensive understanding of gait dynamics and control. This will further contribute to improving the accuracy and relevance of gait simulations.

The physiological muscle coactivation levels could be reproduced with a gait controller trained using a deep RL method that regulated hip, knee, and ankle joint stiffness in a forward-dynamics musculoskeletal-walking simulation. In the vast landscape of RL-based gait research, many studies primarily focus on the accurate simulation of walking motions, often neglecting the intricate dynamics of muscle control theories. However, our investigation revealed an invaluable insight that muscle control strategies that produce substantial coactivations can significantly enhance the stability of walking. The results align with physiological observations. While electromyography-driven and muscle synergy vector-based musculoskeletal simulations focus primarily on reproducing observed muscle activation patterns using a relatively simple musculoskeletal model, our gait simulations allowed to systematically explore and optimize the vast and complex space of muscle activation patterns for desired outcomes, such as energy efficiency or stability.

Our research revealed that simulating muscle coordination to enhance joint stiffness leads to coactivation, potentially reflecting the underlying neural mechanisms of muscle control. Additionally, we showed the importance of considering muscle coactivation patterns in gait simulations. Traditional modeling approaches, which focused on minimizing muscle activations, might underestimate fatigue and joint loading. Notably, high coactivation levels have been frequently observed in populations with mobility challenges, such as the elderly and rehabilitation patients. Addressing coactivation levels would be clinically significant as it can provide insights into the walking dynamics of these specific groups and enhance the relevance and applicability of gait models in clinical aspects. Our approach and findings would increase understanding of the neuronal control of muscles and also increase accuracies in estimating internal body forces along with the forces in the agonist and antagonist muscles.

## Methods

### Data acquisition

The study was approved by the Institutional Review Board of Korea Advanced Institute of Science and Technology. A healthy young adult (male, age: 23 years, weight: 65.4 kg, height: 171.2 cm) without a history of lower limb injury in the last 5 years was included in this study after informed consent was obtained. All methods were performed in accordance with the relevant guidelines and regulations. Reflective body markers were attached to the participant according to the Plug-in Gait full-body marker set protocol from the Vicon system (Oxford, UK). The participant was asked to walk at self-selected normal walking speed. The participant practiced walking for more than 10 min until he could walk naturally. After the practice, the participant walked at his self-selected speed (1.34 m/s), and the trajectories of the body markers were recorded using a motion capture system with 12 cameras (Vantage V5 and MX T-10, Vicon, Oxford, UK).

### Musculoskeletal model

**Figure 4:**
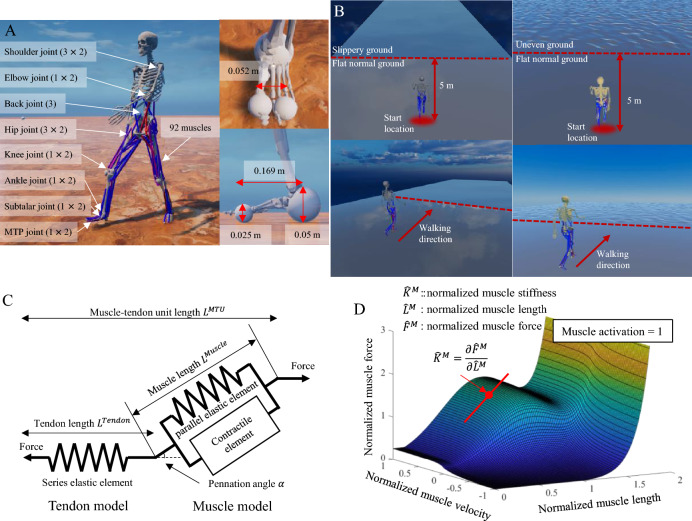
(**A**) A musculoskeletal model with 31 degrees-of-freedom (including 6 DOF of the pelvis) and 92 muscles. (**B**) Ground geometries for gait simulations of slippery ground (left) and uneven terrain (right). (**C**) The Hill type muscle model composed of a muscle contractile element with elastic elements and a tendon model. (**D**) The force–length–velocity surface from our muscle model implementation.

A three-dimensional full-body musculoskeletal model of 31 degrees-of-freedom with 92 muscles was developed to perform a dynamics simulation of gait using the RaiSim dynamics solver v1.1.2^56^ (Fig. 4A). Skeletal geometries, including mass and inertia, and joints types, locations, and rotational axes of a musculoskeletal model, were obtained from a published musculoskeletal model^58^. The knee joint was made as a revolute joint. A custom Hill-type muscle model with an activation dynamics model was developed to provide muscle force for the given muscle length, velocity, and excitation signal^59^. The custom Hill-type muscle model comprised a series combination of tendon as a passive elastic element, and the muscle as both passive elastic and active contractile elements (Fig. 4C). The parameters of muscle–tendon units, such as force–length relationship of the tendon and force–length–velocity relationship of the muscle, were obtained from the Gait2392 model in OpenSim v4.2^8^. Although the published musculoskeletal model^58^ contains its own muscle model and parameters, we opted for the muscle model and parameters in the Gait2392 model due to computational costs of the muscle wrapping. Furthermore, the muscle attachment positions with via-points and activation dynamics parameters were obtained from the same model. The force–length relationship of the tendon and the force–length–velocity relationship of the muscle is shown in Fig. 4C and D. The joints in the lower limb and back of the musculoskeletal model were actuated by 92 muscle models. The upper limb joints, such as the shoulder and elbow joints, were actuated by the stable proportional–differential (PD) control^60^. The stable PD control generated joint torque according to the angular position target, velocity target, and their gain values^31,32^. In this study, the joint velocity target was fixed as 0. The contact bodies in the foot were made of a sphere in the calcaneus and two spheres in the toe (Fig. 4A). The contact and friction forces in the foot were calculated using the RaiSim dynamics solver.

### Reinforcement learning of the standard gait controller

**Figure 5:**
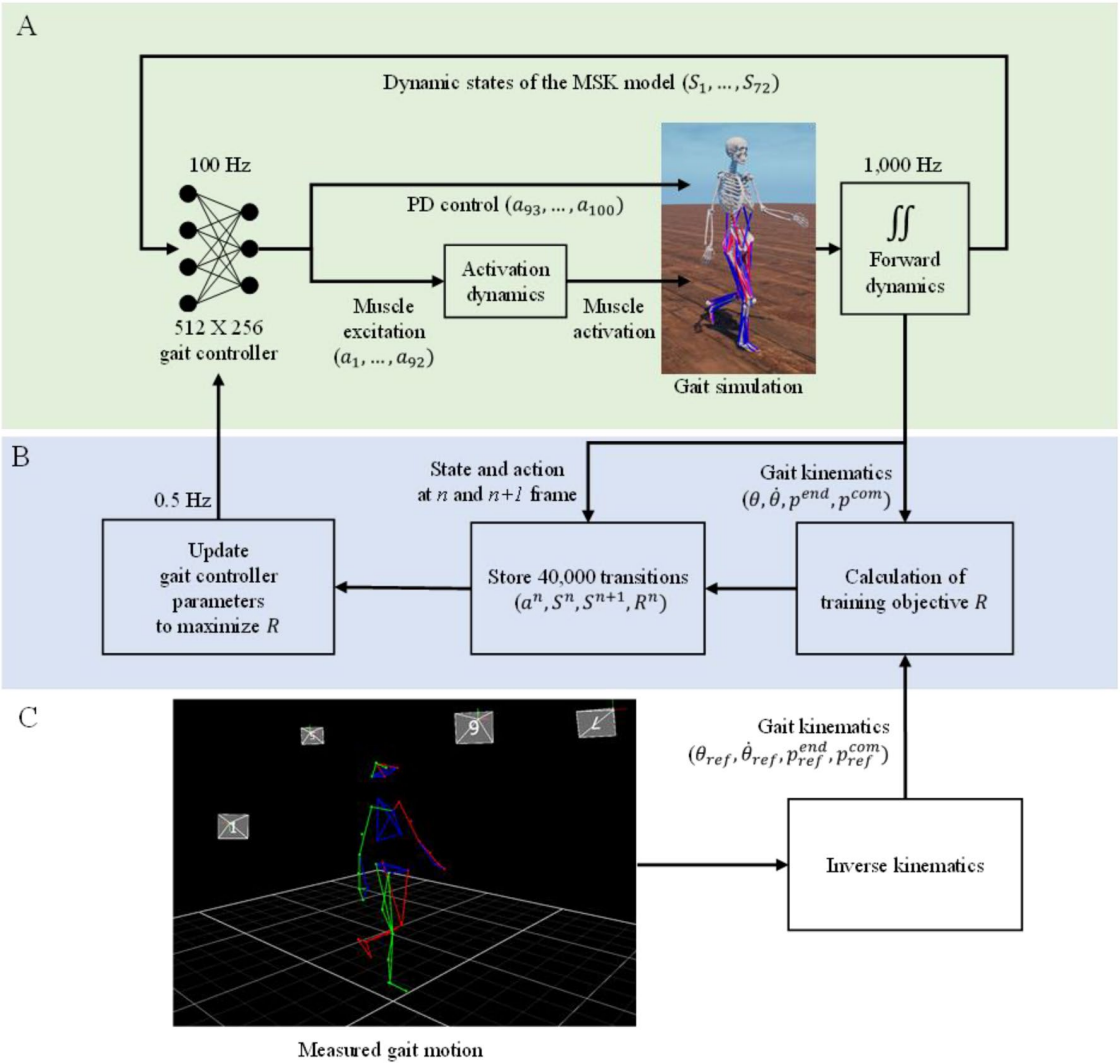
(**A**) Forward dynamics environment for gait simulation using a musculoskeletal model and (**B**) a gait controller and a deep RL environment for training the gait controller using (**C**) measured reference gait motion, where $a, S, R, \theta , \dot{\theta },{p}^{end},$ and ${p}^{com}$ are the action, state, reward, joint angular position, joint angular velocity, end-effector’s positions, and center of mass’s positions, respectively.

A standard gait controller for the three-dimensional full-body musculoskeletal model was developed using a fully-connected deep neural network of two layers with 512 and 256 nodes each (Fig. 5A). The standard gait controller, which generated excitation signals of 92 muscles in the lower limbs and eight position targets of upper limb joints from the dynamic state of the musculoskeletal model, was trained using a deep RL-based imitation learning method^32^ (Fig. 5B and C). The deep RL method collected data transitions about the controller’s input, output, and objective function value to update the gait controller parameters to imitate reference gait kinematics. Specifically, the data transitions comprised the input state of the gait controller at the current frame ($${S}_{n}$$), input state of the gait controller at the next frame ($${S}_{n+1})$$, output action of the gait controller at the current frame ($${a}_{n})$$, and a reward value of the training objective function ($${R}_{n}$$) during the musculoskeletal gait simulation. The proximal policy optimization^61^ was used to implement the deep Rl-based imitation learning method. A total of 40,000 data samples were collected at gait control frequency of 100 Hz during 2.0 s simulation from 200 parallel simulation environments in each update cycle. The samples were used to calculate gradients of the neural network parameters of the standard gait controller. The input state ($$S$$) of the standard gait controller was a vector of 72 values for the dynamic states of the body—orientation of the pelvis (3), rotational and translational velocity of the pelvis (6), height of the pelvis (1), angular position of each joint (25), angular velocity of each joint (25), ground reaction force (6), and center of pressure (6) of each foot with respect to the pelvis frame. The output action ($$a$$) of the standard gait controller was a vector of 100 values for muscle excitation signals of 92 muscles (92), position target of both shoulders (6), and position target of both elbows (2).

To implement the deep RL-based imitation learning method, the body kinematics was calculated from the measured motion capture data of the participant during walking. The lengths and masses of the skeletal model segments were scaled using a static motion capture data. The temporal joint angles of the 20 joints (25 DOF) of the body during one gait cycle were calculated from the motion capture data using the inverse kinematics function of OpenSim. Gait kinematics score ${r}_{track}$, which indicated the performance of the gait controller to make the musculoskeletal model track the reference kinematics, was calculated as a reward value or training objective value of the deep RL-based imitation learning. Gait kinematics score, ${r}_{track}$, was divided into four sub-scores of angular positions, angular velocity, position of center of mass, and end effector (foot, hand, and head) positions according to a previous deep RL-based imitation learning study^32^. In detail, the reward value was calculated as the following equations.

$${r}_{track}= 0.65{r}^{pos}+ 0.1{r}^{vel}+ 0.15{r}^{end}+ 0.1{r}^{com},$$

$${r}^{pos}={\text{exp}}\left[-0.4\left(\sum_{j}{\Vert {\theta }_{ref}^{j}- {\theta }^{j}\Vert }^{2}\right)\right] ,$$

$${r}^{vel}={\text{exp}}\left[-0.000625\left(\sum_{j}{\Vert {\dot{\theta }}_{ref}^{j}- {\dot{\theta }}^{j}\Vert }^{2}\right)\right] ,$$

$${r}^{end}={\text{exp}}\left[-1.25\left(\sum_{e}{\Vert {p}_{ref}^{end}- {p}^{end}\Vert }^{2}\right)\right] ,$$

$${r}^{com}={\text{exp}}\left[-0.5\left({\Vert {p}_{ref}^{com}- {p}^{com}\Vert }^{2}\right)\right] ,$$

where ${\theta }^{j}$ denotes the angle of *j-*th joint; ${\dot{\theta }}^{j}$ denotes the time derivative of ${\theta }^{j}$; ${p}^{end}$ denotes the positions of head, both hands, and both feet; and ${p}^{com}$ denotes the center of the mass position of the musculoskeletal model. The weights of each reward components were determined according to a previous study^32^. The gait controller with a neural network was updated 65,000 times, with 40,000 samples per update, taking about 36 h. We obtained the standard gait controller to track the reference gait kinematics. A frame was selected from the reference motion randomly for training. The joint angles and velocities of the frame were used as initial conditions^32^. The values from the gait controller were used to set both the initial muscle excitations and activations. Initial muscle fiber length and velocity were set as the muscle’s optimal fiber length and zero, respectively. We ran the deep RL training on Linux (Ubuntu 18.04.5 LTS) with the following hardware specifications of CPU: AMD Ryzen Threadripper 3990X 64-Core Processor, RAM: 64 GB, GPU: GeForce RTX 2060 SUPER.

### Fine-tuning of the gait controller for different objectives

Five additional gait controllers with different levels of joint stiffness in the lower limb were created using the fine-tuning method to test the effect of the joint stiffness on muscle coactivation and gait stability. The fine-tuning method in deep RL retrained the previously acquired controller after modifying the reward function. The standard gait controller was retrained with modified reward functions and updated 20,000 times to achieve the pre-determined joint stiffness mimicking the measured gait kinematics (Table 3).

**Table 3:** Objective functions and their weight values for training five different gait controllers.

Reward function (${r}_{total}$)	${r}_{total}= {w}_{track}{r}_{track}+ {w}_{A}{r}_{A}+{w}_{S}{r}_{S};$ ${r}_{track}=0.65{r}_{pos}+0.1{r}_{vel}+0.15{r}_{end}+0.1{r}_{com}$ ${r}_{A}={\text{exp}}\left({\sum }_{i=1}^{\#muscles}-{\left({A}_{i}\right)}^{2}\right) ,{A}_{i}:\mathrm{activation\,of\,}i\mathrm{th\,muscle}$ ${r}_{S}={\text{exp}}\left(-{\left({S}_{Target}^{Hip}-{S}^{Hip}\right)}^{2}-{\left({S}_{Target}^{Knee}-{S}^{Knee}\right)}^{2}-{\left({S}_{Target}^{Ankle}-{S}^{Ankle}\right)}^{2}\right)$					
	${w}_{track}$	${w}_{A}$	${w}_{S}$	${S}_{Target}^{Hip}$(Nm/deg)	${S}_{Target}^{Knee}$(Nm/deg)	${S}_{Target}^{Ankle}$(Nm/deg)
ACT	0.9	0.1	0.0	–	–	–
STIF0	0.9	0.05	0.05	0	0	0
STIF1	0.9	0.05	0.05	5.0	5.0	5.0
STIF2	0.9	0.05	0.05	7.5	7.5	7.5
STIF3	0.9	0.05	0.05	10.0	10.0	10.0

Ss represent target and calculated joint stiffness values.

One fine-tuned controller (controller code: ACT) minimized only the sum of muscle activations squared (activation minimization). The remaining four fine-tuned controllers (STIF0, STIF1, STIF2, and STIF3) had target joint stiffness values for the hip flexion–extension, knee flexion–extension, and ankle plantarflexion–dorsiflexion in addition to the activation minimization in the reward functions. The target joint stiffness values for the three joints were 0.0, 5.0, 7.5, and 10.0 Nm/rad for STIF0, STIF1, STIF2, and STIF3, respectively, as summarized in Table 3. All fine-tuned controller including ACT, STIF0, STIF1, STIF2, and STIF3 were trained on normal flat ground with a friction coefficient of 0.8. The kinematic similarities of the fine-tuned controllers were tested on the same flat ground for 20 s, which included 20 cycles of gait.

We made an additional fine-tuned controller, ACT-random, to understand natural joint stiffness adaptation to slippery ground. ACT-random was trained by fine-tuning the standard gait controller using the same objective function as that of the ACT gait controller but different ground condition whose friction coefficient randomly changed in the range between 0.2 and 0.8 at every gait simulation. The fine-tuning of the gait controller required 20,000 updates or 800 million samples.

### Joint stiffness

Joint stiffness $K$ along the lower limb joint axis was calculated using a force–length relationship of muscle–tendon unit (MTU) and moment arm for the hip flexion–extension, knee flexion–extension, and ankle plantarflexion–dorsiflexion. A partial derivative of joint moment by a muscle with respect to joint angle $\theta$ was calculated for each muscle. The joint stiffness was calculated by summing the partial derivatives of joint moments by all muscles around a joint^62^. For example, the partial derivative of joint moment by the biceps short head (BCS) in the knee can be calculated using the muscle force–length function and the moment arm of the muscle as expressed by the following equations.

$${{\text{K}}}_{{\text{BCS}}}^{{\text{Knee}}}=\frac{\partial \left({r}_{BCS}{\cdot F}_{BCS}\right)}{\partial \theta } =\frac{\partial {r}_{BCS}}{\partial \theta }{\cdot F}_{BCS}+ \frac{\partial {F}_{BCS}}{\partial \theta }\cdot {r}_{BCS}$$

where ${{\text{K}}}_{{\text{BCS}}}^{{\text{Knee}}}$, ${r}_{BCS}$, and ${F}_{BCS}$ represent the knee joint stiffness by BCS, moment arm of BCS about the knee joint, and MTU force of BCS, respectively. The partial derivate of the BCS MTU force with respect to the knee angle can be represented as the following equation using the chain rule where ${L}_{BCS}$ is the MTU length of BCS.

$$\frac{\partial {F}_{BCS}}{\partial \theta }=\frac{\partial {F}_{BCS}}{\partial {L}_{BCS}}\cdot \frac{\partial {L}_{BCS}}{\partial \theta }.$$

Because the muscle moment arm can be obtained from the derivative of the MTU length with respect to the joint angle (${r}_{BCS}= \partial {L}_{BCS}/\partial \theta$)^62^, the knee joint stiffness by BCS can be represented as follows.

$${{\text{K}}}_{{\text{BCS}}}^{{\text{Knee}}}=\frac{\partial {r}_{BCS}}{\partial \theta }{\cdot F}_{BCS}+ {K}_{BCS}^{MTU}\cdot {{r}_{BCS}}^{2},$$

where ${K}_{BCS}^{MTU}$ represents the stiffness of BCS MTU ($\partial {F}_{BCS}/\partial {L}_{BCS}$). The stiffness of BCS MTU is calculated from a series combination of its muscle fiber stiffness (${K}_{BCS}^{M}$) and tendon stiffness (${K}_{BCS}^{T}$) as follows^62^.

$${K}_{BCS}^{MTU}={\left(\frac{1}{{K}_{BCS}^{M}}+\frac{1}{{K}_{BCS}^{T}}\right)}^{-1} .$$

BCS muscle fiber stiffness ${K}_{BCS}^{M}$ is obtained from the slope on the force–length–velocity surface along the muscle length axis for a given muscle state as shown in Fig. 4D. Tendon stiffness ${K}_{BCS}^{T}$ is obtained from the slope of the force–length relationships of the tendon.

The MTU length of BCS can be calculated as a sum of Euclidian distances between the path points of BCS. The BCS has three path points, two on the femur and one on the tibia. The Euclidian distance between the first two path points on the femur is a constant, and the Euclidian distance between the second and third path points is a function of knee flexion angle owing to the movement between the femur and tibia. Therefore, the moment arm of BCS is a function of the knee flexion angle, ${r}_{BCS}= \partial {L}_{BCS}/\partial \theta$, from which the derivative of moment arm with respect to the knee flexion angle, $\partial {r}_{BCS}/\partial \theta$, can be easily calculated.

### Muscle coactivation index

The coactivation indices^4^ of the thigh and shank muscles were calculated for walking using the five different gait controllers. The coactivation index was a value to represent simultaneous firing rate of agonist and antagonist muscles. Four agonist–antagonist muscle group pairs, two for the shank (tibialis anterior–soleus, tibialis anterior–gastrocnemius) and two for the thigh (vastus–biceps femoris, vastus–semitendinosus), were determined with reference to a previous study^4^. Ten muscles, including the vastus intermedius, vastus lateralis, vastus medialis, biceps femoris long head, biceps femoris short head, semitendinosus, tibialis anterior, soleus, medial gastrocnemius, and lateral gastrocnemius, were used for the calculation. The coactivation indices of the four muscle pairs were calculated using the following equation. Here, ${a}_{agonist}$ represents the muscle activation of agonist muscle, and ${a}_{antagonist}$ represents the muscle activation of antagonist muscle.

$$\mathrm{Coactivation index }\left(\mathrm{\%}\right)=100\left(\frac{2\int {\text{min}}\left({a}_{agonist}, {a}_{antagonist}\right)}{\int {a}_{agonist}+\int {a}_{antagonist}}\right).$$

For example, to calculate the coactivation index of the vastus–biceps femoris pair, six coactivation indices were computed for the combinations of three vastus muscles and two biceps femoris muscles. Then, the six coactivation indices were averaged to compute the coactivation index for the vastus-biceps femoris pair. Finally, the thigh coactivation index was obtained by averaging coactivation indices of the two muscle pairs, vastus–biceps femoris and vastus–semitendinosus.

### Gait simulation on slippery ground

Five slippery terrains were created by lowering the ground's friction coefficient from its normal value of 0.8^63^. We used five different friction coefficients: 0.20, 0.25, 0.30, 0.35, and 0.40. The human model initiated its walk from the normal ground with a friction coefficient of 0.8, covered a distance of five meters on this flat ground to stabilize walking dynamics, and then transitioned to the slippery ground as illustrated in Fig. 4B.

Gait simulations using six gait controllers—ACT, SITF0, SITF1, SITF2, STIF3, and ACT-random—were performed on the five slippery ground models. A total of 30 combinations of five ground models and six controllers were used. One gait simulation trial lasted 20 s. The virtual gait trial was repeated 100 times, and the number of falls were obtained (Supplementary Video 1). The foot angle at heel strike and stability margin, which are biomechanical indications for fall risk during walking^40^, were also quantified. The performances of the gait controllers were compared using a Chi-squared test, followed by post-hoc pairwise Chi-squared tests with Bonferroni correction at a significance level of 0.05.

### Gait simulations on uneven terrain

Dynamic gait stabilities of the musculoskeletal models with different gait controllers were tested via gait simulations on uneven terrain. The uneven terrain was randomly generated using the terrain generator function of RaiSim that used the Perlin noise algorithm with a height scale parameter^64^. Four uneven terrain models were developed with four different height scales (0.01, 0.02, 0.03, and 0.04 m) of the terrain generator. The ground model is comprised of both flat and uneven terrains. As in the slippery ground experiment, the human model initiated its walk from the flat terrain, covered a distance of five meters on this flat ground, and then transitioned to the uneven terrain as illustrated in Fig. 4B. A total of 20 combinations of four ground models and five controllers were used.

Each controller underwent a 20-s walking simulation on uneven terrain, repeated 10 times. During the gait simulation, the number of survivals without falls were obtained to quantify the walking stabilities. The performances of the gait controllers were compared using a Chi-squared test, followed by post-hoc pairwise Chi-squared tests with Bonferroni correction at a significance level of 0.05.

### Supplementary Information

Supplementary Legends.

Supplementary Video 1. It provides video summary of the study and demonstrations of the simulation results.

## Data Availability

The datasets used and/or analyzed during the current study are available from the corresponding author on reasonable request.
