# Supplementary material for: Higher coactivations of lower limb muscles increase stability during walking on slippery ground in forward dynamics musculoskeletal simulation
Source: Sci Rep. 2023 Dec 20;13:22808. doi: 10.1038/s41598-023-49865-w (PMC10739792; doi:10.1038/s41598-023-49865-w)
Supplement: Supplementary file 1 — Supplementary Legends. [file 41598_2023_49865_MOESM1_ESM.docx]

Video 1. It provides video summary of the study and demonstrations of the simulation results.
